# B cell subset alteration and the expression of tissue homing molecules in dengue infected patients

**DOI:** 10.1186/s12929-018-0467-8

**Published:** 2018-08-27

**Authors:** Kovit Pattanapanyasat, Ladawan Khowawisetsut, Ampaiwan Chuansumrit, Kulkanya Chokephaibulkit, Kanchana Tangnararatchakit, Nopporn Apiwattanakul, Chonnamet Techasaensiri, Premrutai Thitilertdecha, Tipaporn Sae-Ung, Nattawat Onlamoon

**Affiliations:** 1 Biomedical Research Incubator Unit, Research Group and Research Network Division, Research Department, Faculty of Medicine Siriraj Hospital, Mahidol University, Bangkok, Thailand; 2 Department of Parasitology, Faculty of Medicine Siriraj Hospital, Mahidol University, Bangkok, Thailand; 3 Department of Pediatrics, Faculty of Medicine Ramathibodi Hospital, Mahidol University, Bangkok, Thailand; 4 Department of Pediatrics, Faculty of Medicine Siriraj Hospital, Mahidol University, Bangkok, Thailand; 5 Research group in Immunobiology and Therapeutic Sciences, Faculty of Medicine Siriraj Hospital, Mahidol University, 2 Wanglang Road, Bangkoknoi, Bangkok, 10700 Thailand; 6 Master of Science program in Immunology, Department of Immunology, Faculty of Medicine Siriraj Hospital, Mahidol University, Bangkok, Thailand

**Keywords:** Antibody secreting cells, Trafficking molecules, Severity, Dengue

## Abstract

**Background:**

B cells play an essential role during dengue viral infection. While a major expansion of antibody secreting cells (ASCs) was observed, the importance of these increased frequencies of ASCs remains unclear. The alteration of B cell subsets may result from the expression of tissue specific homing molecules leading to their mobilization and distribution to different target organs during acute dengue viral infection.

**Methods:**

In this study, whole blood samples were obtained from thirty pediatric dengue-infected patients and ten healthy children and then stained with fluorochrome-conjugated monoclonal antibodies against CD3, CD14, CD19, CD20, CD21, CD27, CD38, CD45, CD138 and homing molecules of interest before analyzed by polychromatic flow cytometry. B cell subsets were characterized throughout acute infection period.

**Results:**

Data shows that there were no detectable differences in frequencies of resting, activated and tissue memory cells, whereas the frequency of ASCs was significantly increased and associated with the lower frequency of naïve cells. These results were found from patients with both dengue fever and dengue hemorrhagic fever, suggesting that such change or alteration of B cells was not associated with disease severity. Moreover, several homing molecules (e.g., CXCR3 and CCR2) were found in ASCs, indicating that ASCs may distribute to inflamed tissues and various organs.

**Conclusions:**

Findings from this study provide insight into B cell subset distribution. Furthermore, organ mobilization according to homing molecule expression on different B cell subsets during the course of dengue viral infection also suggests they are distributed to inflamed tissues and various organs.

## Background

Varied clinical outcomes are one of the hallmarks of dengue viral infection. The outcomes range from aymptomatic infection to infection that can result in mild fever (dengue fever or DF) or severe hemorrhagic fever (dengue hemorrhagic fever or DHF) and dengue shock syndrome (DSS) [1]. The major characteristic symptoms of DSS are hemorrhagic phenomenon (e.g., petechiae, mild mucous membrane or skin bleeding) and shock [2, 3]. The dengue virus results in 50–100 million infections leading to 500,000 hospitalizations and > 20,000 fatal cases per year worldwide as estimated by the World Health Organization (WHO) [4–6]. The dengue virus is transmitted primarily by a bite from an infected female mosquito, *Aedes aegypti*. The infection by dengue virus occurs in humans of all ages. Although a marked increase in a number of adult with severe dengue was also observed in countries such as Taiwan, Singapore and Sri Lanka, the highest rates of severe dengue occur in children from some countries such as Thailand and Viet Nam [7].

There are four serotypes of dengue including DENV-1, DENV-2, DENV-3 and DENV-4 [8] that express both serotype unique and cross reactive epitopes. After primary DENV infection, recovered patients generate potent antibody responses that to a large extent cross react with the 4 serotypes. However, homologous reinfection does not occur and whether antibodies are responsible for this protection is not fully known. Patients that are re-infected with the different serotype (heterologous) not only remain susceptible to infection with the heterologous dengue virus but in select cases show an increased susceptibility to developing a severe form of the disease termed dengue hemorrhagic fever (DHF) and dengue shock syndrome (DSS). While still considered controversial, the phenomenon is termed antibody mediated enhancement (ADE) [9–12].

B cells have been shown to play a major role during infection with dengue viruses highlighted by the recent observation of a significantly high number of plasmablast/plasma cells that appear during acute dengue infection [13–16]. Activation of B cells through dengue-specific B cell receptor (BCR) has been reasoned to induce B cell proliferation and differentiation into effector plasma cells or long lived memory B cells [17]. The antibody secreting cells (ASCs), which is refer to a combination of both plasmablasts and plasma cells, produced antibodies which have an important role not only in the protection against subsequent exposure [18] but can also lead to an increase in the risk of infection in some cases [19].

The objectives of the present study were to characterize in detail changes in the B cell subpopulations and plasmablasts/plasma cells during acute dengue infection and to identify alterations in the expression of trafficking molecules by the different B cell subsets. It was reasoned that the identification of unique set of homing markers by cells in these patients with the severe forms of the disease may provide clues to the pathogenic mechanisms that distinguish asymptomatic from DHF/DSS. The results of this study are the basis of this report.

## Methods

### Study population and sample collection

In this study, 30 dengue infected children and 10 healthy, age-matched children were recruited from the Faculty of Medicine Siriraj Hospital and Faculty of Medicine Ramathibodi Hospital, Mahidol University, Bangkok, Thailand. The patients were categorized into dengue fever (DF), dengue hemorrhagic fever (DHF) based on the 1997 WHO classification of dengue infection which has been currently acceptable for clinical practice in Thailand. Information about patient cohort is detailed in Table 1 while the clinical features of patients are also shown in Table 2. The blood samples were collected aseptically by venipuncture into a sterile 3.2% sodium citrate blood collection tube and immediately transported to the laboratory and stored at room temperature (RT) until ready for flow cytometric analyses.

**Table 1:** Summary of study subjects

Characteristic	DF	DHF	Healthy individuals
Total number of samples	10	20	10
Number of males/number of females	3/7	6/14	5/5
Age (year)	5–14	5–19	10–19
Number of patients infected with dengue virus serotype:			
1	5	4	–
2	1	3	–
3	4	9	–
4	–	2	–
1 / 2	–	1	–
1 / 4	–	1	–

**Table 2:** Clinical feature of dengue infected patients

Patient ID	Category	Clinical feature
V001	DF	No evidence of leakage.
V002	DF	Hematocrit rising 14%, evidence of pleural effusion not noted.
V003	DF	Hematocrit rising 10%, evidence of pleural effusion not noted.
V004	DF	No evidence of leakage.
V005	DF	No evidence of leakage.
V006	DF	No evidence of leakage, hypermenorrhea.
V007	DF	Hematocrit rising 8% (from 37 to 40%).
V008	DF	No evidence of leakage.
V009	DF	No evidence of leakage.
V010	DF	No evidence of leakage.
V011	DHF I	No bleeding, there was evidence of hemoconcentration, right pleural effusion by physical examination and chest X-ray.
V012	DHF I	Decrease breath sounds in the right lung but negative chest X-ray, hemoconcentration from 38 to 43%.
V013	DHF I	Hematocrit rising 14%, evidence of pleural effusion not noted.
V014	DHF I	Evidence of minimal right pleural effusion.
V015	DHF I	Minimal right pleural effusion, decrease breath sound right lung.
V016	DHF I	Mild dehydration and hypokalemia.
V017	DHF I	Mild dehydration
V018	DHF I	Moderated dehydration.
V019	DHF I	Hyponatremia with mild dehydration.
V020	DHF I	Mild dehydration
V021	DHF I	Mild dehydration
V022	DHF I	Right pleural effusion.
V023	DHF I	Mild dehydration
V024	DHF II	Hematocrit rising from 35 to 42%, lowest Hematocrit being 32.8%, epistaxis, chest X-ray with right pleural effusion.
V025	DHF II	Right pleural effusion.
V026	DHF III	Narrow pulse pressure, hemoconcentration, chest X-ray with right pleural effusion.
V027	DHF III	Evidence of hypotension but not profound shock.
V028	DHF III	Hemoconcentration and hypotension, underlying disease of b-thalassemia, pleural effusion from physical examination and chest X-ray.
V029	DHF III	Hemoconcentration, hypoalbuminemia, vaginal bleeding, epistaxis, narrow pulse pressure, chest X-ray with pleural effusion.
V030	DHF III	Hypokalemia.

### Monoclonal antibodies and reagents

Fluorochrome-conjugated monoclonal antibodies against a variety of cell surface molecules using for the phenotypic characterization of B cell subsets included anti-CD3 conjugated with phycoerythrin-cyanine 7 (PE-Cy7), anti-CD14 conjugated with allophycocyanin- cyanine 7 (APC-Cy7), anti-CD19 conjugated with brilliant violet 510 (BV510), anti-CD20 conjugated with alexa fluor 700 (A700), anti-CD21 conjugated with allophycocyanin (APC), anti-CD27 conjugated with brilliant violet 605 (BV605), anti-CD38 conjugated with brilliant violet 421 (BV421), anti-CD45 conjugated with peridinin chlorophyll protein (PerCP), anti-CD138 conjugated with fluorescein isothiocyanate (FITC). PE-conjugated monoclonal antibodies against a variety of cell surface specific homing molecule that have previously been reported to facilitate the migration of cells to different tissues or organs were utilized and included antibodies against CCR2, CCR7, CCR9, CCR10, CD29, CD62L, CD103, CD122, CD132, CD137, CXCR3, CD278, β7 integrin and CXCR4.

### Immunofluorescent staining and flow cytometric analysis

Aliquots of whole blood samples in a volume of 100 µL were stained with a pre-determined optimal concentration of a cocktail of fluorochrome-conjugated monoclonal antibodies against cell surface molecules. These included CD3, CD14, CD19, CD20, CD21, CD27, CD38, CD45, and CD138. Each aliquot was then stained with, either CCR2, CCR7, CCR9, CCR10, CD29, CD62L, CD103, CD122, CD132, CD137, CXCR3, CD278, β7 integrin or CXCR4. The stained samples were incubated for 15 min in the dark at RT. After incubation, 2 mL of 1X FACS lysing solution was added to each tube and the tubes incubated for 10 min in the dark at RT to lyse red blood cells. The samples were then washed in phosphate buffered saline (PBS). The supernatant fluid was discarded and the stained cells were re-suspended in 300 μL of PBS. Stained samples were stored at 2 °C to 8 °C until ready for analysis (less than 8 h after blood collection). The stained samples were analyzed on an LSRFortessa flow cytometer by using FACSDiva software (BD Bioscience, San Jose, CA). Data analysis was performed using FlowJo software (Tree Star, Ashland, OR).

### Statistical analysis

GraphPad Prism 5.0 (GraphPad software) was used for statistical analysis. Mann-Whitney U test and 1-way ANOVA followed by Bonferroni’s multiple comparisons test were used for unpaired analysis for comparisons among the groups of data sets. The data for each comparative analysis was calculated as mean ± SD. *P*-values < 0.05 were considered statistically significant.

## Results

### Identification of changes in B cell subsets during acute dengue infection

**Fig. 1:**
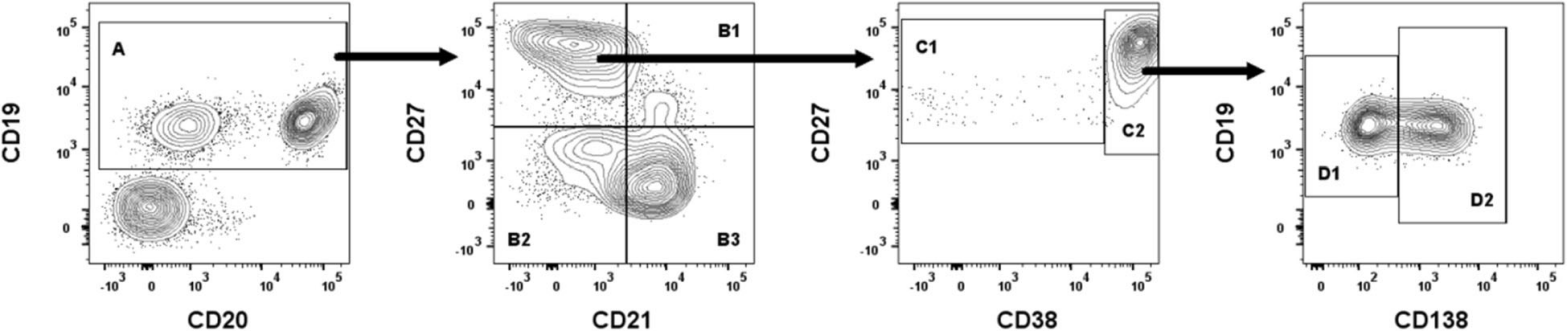
A representative gating strategy used to define B cell subsets. Total B cells (A; CD19+CD20-, CD19+CD20+) were identified into resting memory B cells (B1; CD21+CD27+), tissue memory B cells (B2; CD21-CD27-), naive B cells (B3; CD21+CD27-), activated memory B cells (C1; CD21-CD27+CD38−/low), ASCs (C2; CD21-CD27+CD38high), plasmablasts (D1; CD27highCD38highCD138-), and plasma cells (D2; CD27highCD38highCD138+) in dengue patient

We first sought out to characterize changes in B cell subsets, in the blood samples from patients with acute DENV infection, to establish a foundation for our studies aimed at defining the tissue and organ homing molecules on these subsets. The overarching rationale for this study was that knowledge of the homing patterns of the subsets may help distinguish the severe forms of dengue. The gating strategy utilized for the identification of B cell subsets is illustrated in Fig. 1. As noted, the gated population of CD19 and CD20 expressing lymphoid cells was utilized as a marker for total B cells. The differential expression of CD21, CD27, CD38 and CD138 by the gated population of CD19+/CD20+ B cells was then utilized to distinguish the B cells into 6 subpopulations. The CD21+CD27- subset was identified as naïve B cells and CD21+CD27+ was considered as resting memory B cells, while CD21-CD27- was considered as tissue memory B cells. The CD21-CD27+ subset was further analyzed based on the expression of CD27 and CD38 to distinguish activated memory B cells from ASCs. The activated memory B cells were identified as cells expressing CD21-CD27+CD38−/low and the ASCs were identified as cells that were CD21-CD27+CD38high. The ASC populations, in turn, were further distinguished into plasmablasts that were identified based as cells expressing CD27highCD38highCD138- whereas plasma cells were identified based on the expression of CD27highCD38highCD138+ .

**Fig. 2:**
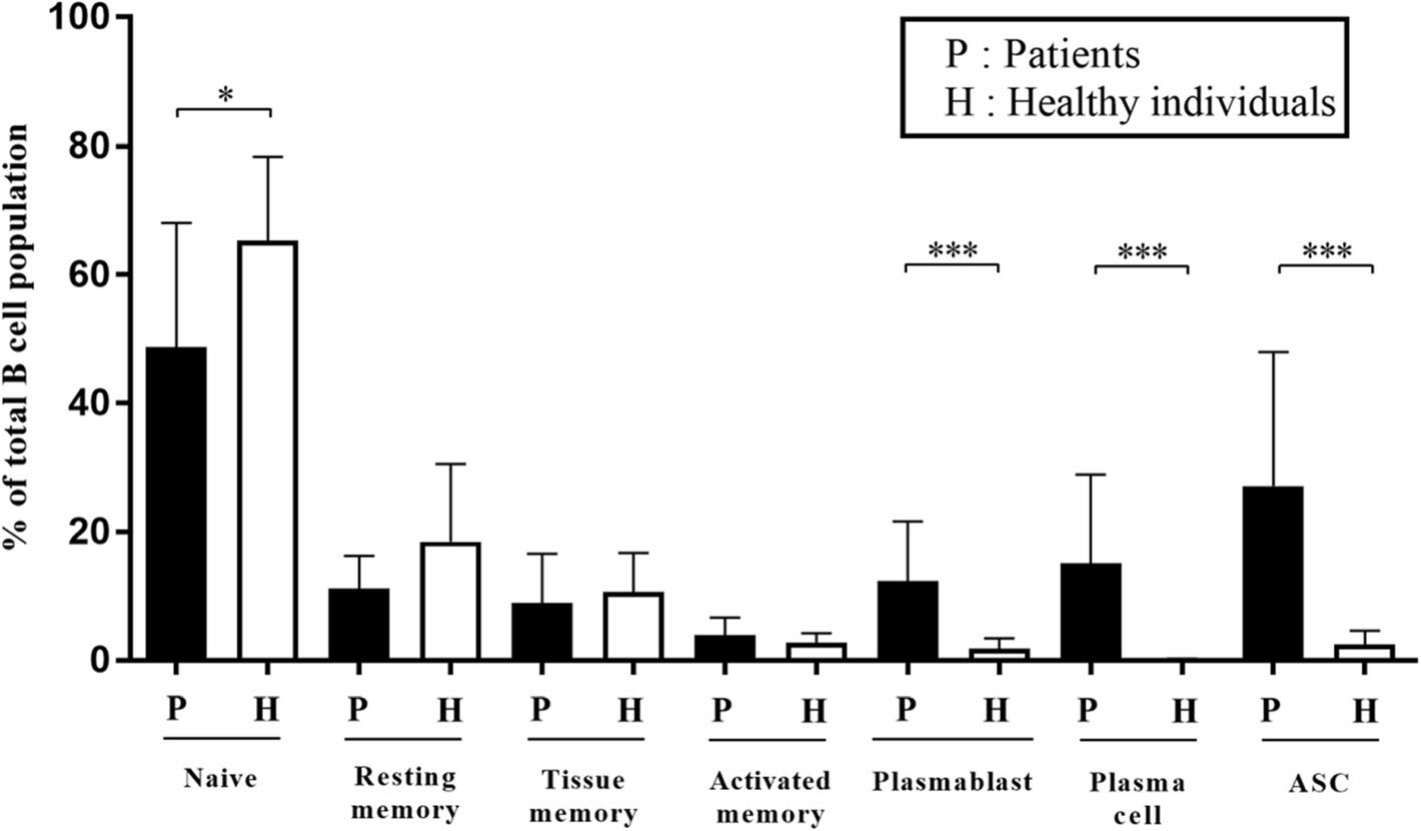
Comparisons of B cell subsets during acute DENV infections. High level of ASCs was observed in dengue infected patients. Significantly lower frequencies of naïve and resting memory B cells were observed in patients when compare with healthy individuals (*p < 0.05, by Mann-Whitney U test). Significantly higher frequencies of plasmablasts/plasma cells or ASCs were observed in patients when compare with healthy individuals (***p ≤ 0.0005, by Mann-Whitney U test), whereas activated memory and tissue memory B cells were not significantly different when compare between patients with dengue and healthy individuals

The frequencies of B cell subpopulations in samples (*n* = 30) from dengue patients were compared with samples from healthy individuals (*n* = 10). As seen in Fig. 2, the samples from the dengue patients showed a lower percentage of the naïve B cell subset and a high percentage of ASC populations as compared with the control samples. When further identifying ASC subsets into plasmablasts and plasma cells, both of them from the dengue patients still showed significantly higher percentages when compared to healthy individuals. These changes are consistent with our previous reports of changes in subsets of B cells during acute dengue infection [15]. The other B cell subsets including resting memory, tissue memory and activated memory were presented in low frequencies and there were no significant differences in these subsets between samples from the dengue patients and controls. We next analyzed these data in efforts to determine if the degree of changes in the B cell subsets noted were correlated with the severity of DENV infection in these patients. Interestingly, no significant difference in any B cell subpopulations were noted in samples from DF and DHF patients (data not shown).

### Kinetics of different B cell subsets during acute DENV infection

**Fig. 3:**
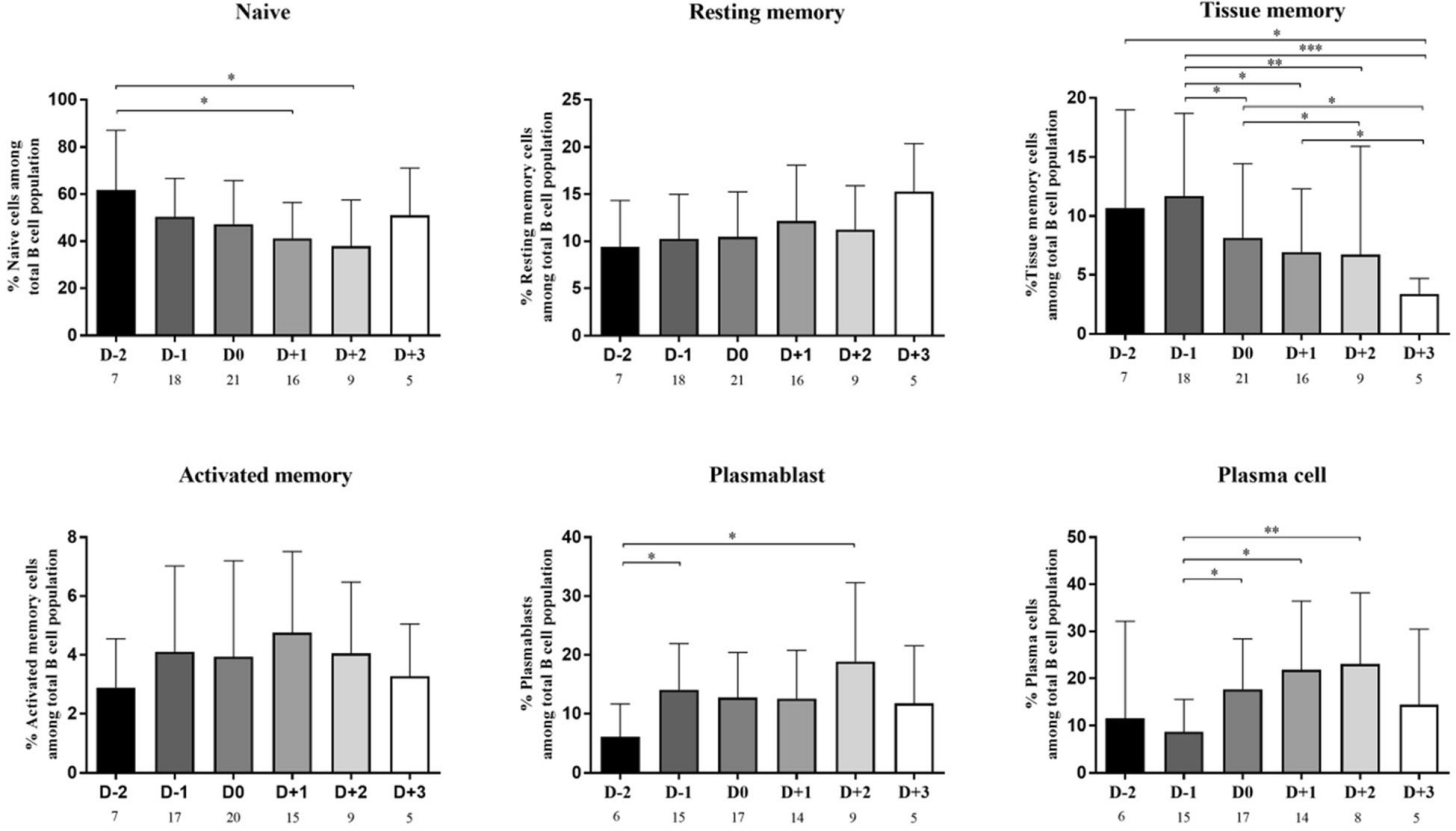
Percentage of B cell subsets at different time points during acute DENV infection. Values (*n*) indicate numbers of patients for each time point. The percentage of naïve and tissue memory B cells had started decreasing from D-2 and D-1 to D+2 and D+3, respectively, whereas plasma cells started to increase from D-1 and to D+2. Other subsets of B cell including resting memory, activated memory and plasmablast showed no specific pattern. Only tissue memory showed significantly lower frequencies at D+2 and D+3 when compare with D-1 (*p < 0.05, ** *p* < 0.005 and ***p ≤ 0.0005 by Mann-Whitney U test)

Since significant changes of B cell subsets occur during acute dengue infection, it was reasoned that the frequency of each B cell subset may differ based on the kinetics of infection. To address this issue, the results of the B cell subsets were compared in relation to day of defervescence (D0) which was defined as the date when the fever dropped below 37.5 °C and remained so for 48 h. The day prior to defervescence was defined as day minus 1 (D-1) whereas the day after defervescence was defined as day plus 1 (D+1). The kinetics of B cell subsets in samples from day D-2 to +3 of defervescence were thus examined. Since the evaluated data from each patient was not complete for the entire period, the data from each available time point were pooled and the number of patients that were evaluated on each time point was indicated. As seen in Fig. 3, there appeared to be a gradual decrease in the average percentage of naïve B cells at D-2 to +3 giving values of 61.8%, 50.3%, 47.2%, 41.2%, 37.8% and 51.0%, respectively. The average percentage of resting memory B cells at D-2 to D+3 while not statistically significant showed a trend towards an increase with values of 9.4%, 10.3%, 10.5%, 12.2%, 11.3% and 15.3%, respectively. On the other hand, there was clearly a significant decrease in the frequencies of tissue memory B cells from D-2 to +3 with values of average percentage of 10.7%, 11.7%, 8.1%, 6.9%, 6.7% and 3.4%, respectively. The average percentages of activated memory B cells at D-2 to +3 did not show an appreciable change. When the data for the frequencies of ASCs were analyzed, the average percentages of plasmablasts at D−2 to +3 were 6.2%, 14.1%, 12.7%, 12.6%, 18.9% and 11.8%, respectively, whereas the average percentages of plasma cells at D-2 to +3 were 11.6%, 8.7%, 17.7%, 21.8%, 23.1% and 14.5%, respectively. Thus, a significant increase in the frequency of ASCs as a function of time was noted for these subsets.

We also determined whether there was any difference between B cell subset responses based on the phases of disease. Three different phases were identified including the febrile phase (D-2 and D-1), the defervescence phase (D0 and D+1) and the afebrile phase (D+2 and D+3) as shown in Table 3. We found that there was a gradual decrease in the average frequency of naïve B cells with values of 53.3%, 44.6% and 41.8% associated with the febrile, defervescence and afebrile phases, respectively. While the average percentage of resting memory and activated B cells did not show significant changes based on the phases of the diseases, the average percentage of tissue memory B cells associated with febrile, defervescence phase and afebrile phases gave values of 11.4%, 7.6% and 5.7%, respectively, denoting a significant decrease as a function of disease phase. The average percentages of plasmablasts and plasma cells also progressively increased from the febrile phase and defervescence phase to the afebrile phase with values of 12.0%, 12.7% and 16.9% for the plasmablasts and 9.5%, 19.5% and 20.7%, respectively for the plasma cells. The results for the levels of the other subsets showed no difference in any phase of infection. Moreover, analyses of these data based on the different phases did not show any difference based on the severity (DF versus DHF/DSS) of DENV infection.

**Table 3:** Comparison of B cell subsets between different phases during acute infection

Acute infection phases	Frequency (%)					
	Naïve B cells	Resting memory B cells	Tissue memory B cells	Activated memory B cells	Plasmablasts	Plasma cells
Febrile (D-2 and D-1)	52.7 ± 19.9*	9.9 ± 4.4	11.0 ± 7.0*^,^**	3.8 ± 2.8	12.6 ± 8.2	10.1 ± 11.8*^,^**
Defervescense (D0 and D+1)	44.4 ± 18.2	12.1 ± 5.3	7.5 ± 6.3***	4.4 ± 3.2	12.0 ± 8.1	19.3 ± 12.8
Afebrile (D+2 and D+3)	43.4 ± 21.4	13.7 ± 4.9	5.8 ± 9.2	3.6 ± 1.9	15.0 ± 13.6	18.5 ± 16.5

Results are showed as mean percentages ± standard deviation. *indicates *p* < 0.05 when compare to defervescence phase, **indicates *p* < 0.05 when compare to afebrile phase and ***indicates *p* < 0.05 when compare between defervescence and afebrile phase

### Comparative analysis of the tissue/organ homing molecule expression on B cell subsets

**Fig. 4:**
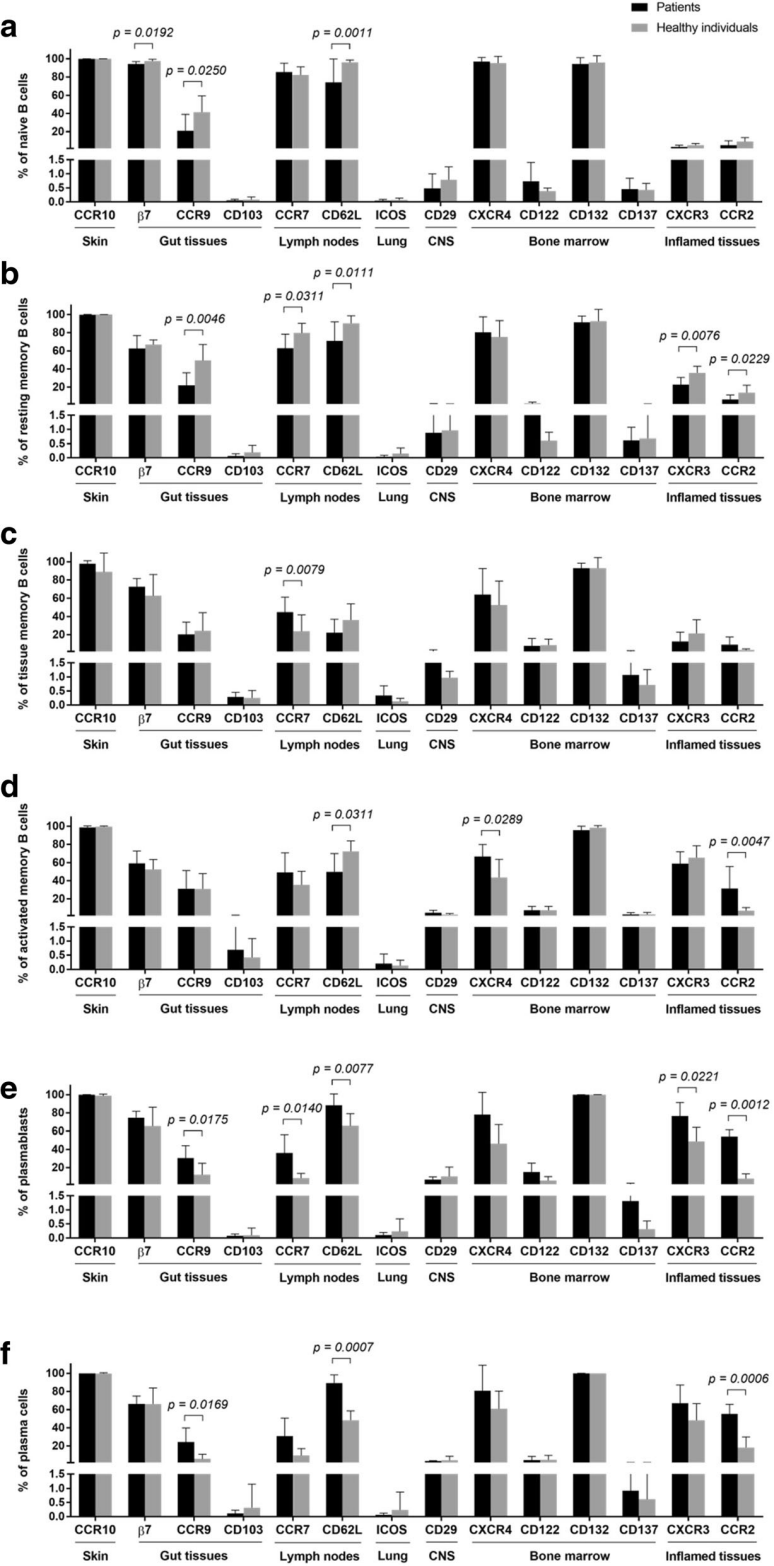
Comparison of specific organ homing molecules of B cell subsets. Frequencies of B cell subsets including (**a**) naïve B cells, (**b**) resting memory B cells, (**c**) tissue memory B cells, (**d**) activated memory T cells, (**e**) plasmablasts and (**f**) plasma cells, were observed for their specific expressions to different organs; skin (CCR10), gut tissues (β7, CCR9, and CD103), lymph nodes (CCR7 and CD62L), lung (ICOS), CNS (CD29), bone marrow (CXCR4, CD122, CD132, and CD137), and inflamed tissues (CXCR3 and CCR2). Changes in frequencies of B cell subpopulations expressing individual marker were compared between dengue-infected patients and healthy donors. Significant differences are indicated when *p*-values < 0.05 by 1-way ANOVA followed by Bonferroni’s multiple comparisons test

The precise mechanisms that lead to DHF/DSS in a subset of dengue infected patients has been a subject of intense investigation by a number of laboratories for quite some time and remains to be defined. We hypothesized that an examination of cell surface molecules that promote the trafficking of B cells to specific tissue/organs may provide clues as to the site at which the host immune response is focused during acute infection. We thus selected to study cell surface molecules that promote cells to traffic to the skin (CCR10), gut tissues (β7, CCR9 and CD103 or αEβ7), lymph nodes (CCR7, CD62L), lung (CD278), central nervous system (CD29), bone marrow (CXCR4, CD122, CD132, CD137) and/or inflamed tissues (CXCR3, CCR2). We studied the expression of these homing markers on different B cell subsets in blood samples from healthy subjects (*n* = 8) and dengue infected patients (*n* = 9). A summary of the data on these markers on the various B cell subsets in samples from dengue patients as compared with healthy subjects are illustrated in Fig. 4.

While significant differences in the frequencies of subsets that express a variety of these markers was noted, it was reasoned that a focus on those markers that were increased in samples from dengue infected patients as compared with healthy controls would be more informative to begin with than those that decreased with the prejudice that such increases would suggest increased trafficking to the specific tissue/organ. What appears clear is that the most notable increases in homing markers appeared to be expressed by plasmablasts and plasma cells from the dengue infected patients as compare with healthy controls. Thus, significant increases were noted in the frequencies of plasmablasts and plasma cells from dengue patients that expressed CCR9 (homing to the small intestine), CCR7 and CD62L (lymph node homing), CXCR4 (bone marrow homing) and markers such as CXCR3 and CCR2 that are associated with homing to inflammatory sites. These findings suggest that there is a greater degree of mobilization of the plasmablasts and plasma cells to the small intestine, lymph nodes and the bone marrow that serve as sites where the inflammatory response to dengue is apparently occurring. Increased frequencies of tissue memory B cells that expressed CCR7 and activated memory B cells that expressed CXCR4 and CCR2 were also noted but the significance of these findings remain unclear. In contrast, significant decreases in the frequencies of naïve B cells that expressed CCR9 and CD62L and resting memory B cells that expressed CCR9, CCR7 and CXCR3 as well as activated memory B cells that expressed CD62L in blood samples from the dengue infected patient as compared with healthy controls were also noted. The significance of such decreases in dengue infected patients is not clear at present.

The expression of homing molecules were also compared between different B cell populations that were observed in dengue infected patients. When compared to naïve B cells, lower frequencies of plasmablasts and plasma cells that expressed β7 (*p* = 0.0224 and 0.0003, respectively) and CCR7 (*p* < 0.0001) were observed. The frequency of plasma cells that expressed CCR7 was also lower than resting memory B cells (*p* = 0.0154). In contrast, higher frequencies of plasmablasts and plasma cells that expressed CD62L were observed when compared to tissue memory (*p* < 0.0001) and activated memory (*p* = 0.0060 and 0.0044, respectively) B cells. Frequencies of plasmablasts and plasma cells that expressed CXCR3 and CCR2 were also higher than naïve, resting memory and tissue memory B cells (*p* < 0.001). Moreover, higher frequencies of plasmablasts and plasma cells that expressed CCR2 were observed when compared to activated memory B cells (*p* = 0.0160 and 0.0063, respectively). Significant changes in the frequencies of activated memory B cells that express homing molecules were also observed. When compared to naïve B cells, lower frequencies of activate memory B cells that expressed β7 (*p* < 0.0001) and CCR7 (*p* = 0.0010) were observed. In addition, when compared to activated memory B cells, lower frequencies of naïve, resting memory and tissue memory B cells that expressed CD29 (*p* = 0.0003, 0.0011 and 0.0126, respectively), CXCR3 (*p* < 0.0001) and CCR2 (*p* = 0.0008, 0.0017 and 0.0072, respectively) were observed. Frequencies of activated memory B cells that express CD137 were also higher than naïve (*p* = 0.0088) and resting memory (*p* = 0.0197) B cells. Taken together, the results showed that B cell populations with activated phenotype (activated memory B cells, plasmablasts and plasma cells) had higher ability to mobilized to inflamed tissues than cells in resting stage (naïve, resting memory and tissue memory B cells).

## Discussion

Several studies have previously documented the findings that dengue virus infection results in marked increases in B cells, especially plasmablasts, plasma cells or antibody-secreting cells (ASCs) [14–16, 20]. The results reported herein confirm these previous findings. While there is a reason to suspect that healthy individuals living in dengue endemic areas might have high or equal level of activated B cells when compare to acute infected patients as a result of previous exposure or an inapparent dengue infection, low levels of plamablasts, plasma cells and activated memory B cells in healthy subjects as observed in this study suggests that dengue exposure must reach a certain threshold in order to induce B cell responses. The natural infection might be required to maintain sufficient levels of antigen exposure as suggested by a study showing that a long term dengue immune memory after vaccination with a chimeric tetravalent DENV vaccine appears relatively low when compare to individuals with a history of natural infection [21]. As reported earlier, the activated B cells proliferate and differentiate into substantial numbers of antibody-secreting cells to produce soluble antibodies against a wide variety of dengue viral epitopes, some of which specifically recognize and neutralize the dengue virus [14, 22]. Amongst these antibodies are also antibodies that are potentially involved in the enhancement of severe disease including DHF or DSS in secondary infection or re-infection with the different type of the dengue virus [23]. Therefore, during acute dengue infection, a large number of plasmablasts, plasma cells or ASC are produced in response to invading foreign pathogens. Moreover, our study also shows that there is a decrease in the frequencies of naïve B cells in dengue infected patients, which is consistent with a previous report of dengue patients in northeast Brazil that showed the decreased proportion of naive and resting memory B cells [20]. Their study also showed that B cells in individuals with severe secondary DENV infection were induced to undergo apoptosis by the expression of the pro-apoptotic marker CD95 or Fas receptor (FasR). Thus apoptotic cell death may be promoted through the engagement of the CD95/CD95L or FasR/ FasL pathway [20, 24–26]. These data suggest that decreased naive and resting memory B cells in patients maybe secondary to apoptosis that is induced by dengue virus [27, 28].

Several reports on B cell subsets during dengue infection have been published. One such report showed interestingly that the frequencies of plasmablast were significantly higher in patients following secondary DF and complicated dengue fever (DFC) infection than during primary dengue infection [20]. A study of pediatric dengue patients in Nicaragua reported that the frequencies of plasmablasts/plasma cells was not significantly different in samples from DF as compared with patients with DHF/DSS [16] that is similar to the results of the studies we report herein. Other studies conducted on dengue patients in Brazil and Singapore studied the kinetics of the plasmablast response and reported that peak plasmablast were observed between days 4–7 of dengue symptoms [14, 20]. Similarly, a report of dengue patients in Thailand found that peak plasmablasts were observed in dengue patients at day 6 or 7 after onset of symptoms [15]. While these data showed plasmablast response at different days after the beginning of fever symptom, our study observed the kinetics of B cell subsets at D-2 to +3 of defervescence. We found that while the frequencies of plasma cells and ASCs were low during the febrile phase (D-2 or −1), these frequencies gradually increased during the afebrile phase (D+2 or +3). In contrast, naïve and tissue memory B cells showed high numbers during the febrile phase (D-2 or −1) that gradually decreased during the afebrile phase (D+2 or +3). Other subsets of B cell including resting memory, activated memory and plamablasts showed similar levels at different time points of infection. Therefore, our results suggest that in the early stage of infection, naïve and tissue memory B cells encounter dengue virus and become activated. These activated B cells develop into plasmablasts/plasma cells or ASCs to produce antibodies which are then specifically eliminated during the late stages of infection. However, comparisons of B cell subset responses in patients with DF and DHF at different time points of infection showed that none of the changes in B cell subsets were associated with the severity of infection. Interestingly, while both plasmablasts and plasma cells contribute to a pool of ASCs, the kinetic levels of these 2 populations are differences as plasmablasts relatively maintain their levels whereas plasma cells decline in a later period. Data indicate that blood circulating plasmablasts derived from activated memory B cells whereas it developmental fate to become either short-lived plasmablasts or long-lived plasma cells remain unclear [29]. However, the decline in plasma cell level suggested the migration of these blood circulating plasma cells to their survival niches such as bone marrow which is crucial for the generation of long-lived plasma cells [30]. It is also unclear how these ASCs contribute to the maintenance of dengue-specific antibody in the serum especially in people from endemic area that may have a repeat exposure to the dengue virus.

B cells play an important role in adaptive immune system by secretion of antigen specific antibodies that contribute to protection and response against the invading pathogens. During the course of infection, effector B cells are mobilized to different organs or tissues in response to chemokine gradients that are generated by cells at sites of infection. The type of cells within a given infected tissue synthesize distinct set of chemokines and the effector cells expressing the receptor for the chemokine set up a pattern of migration that leads to the trafficking of cells to the site of infection. The migration of effector B cells into the infected areas thus mainly depends on adhesion molecules, homing receptors and chemokines. To understand B cell responses in different location, it is important to determine homing markers expressed on B cells, locations that these homing markers guide B cell to go to and the function of B cells at the site of infection. Therefore, we examined homing molecules that express on different B cell subsets that may lead to the migration of these B cells to different tissues or organs where they are optimally suited to deal with particular type of infections. We thus included the analysis of different homing markers that included CCR10 (home to skin) [31, 32], β7 integrin, CCR9 and CD103 (home to gut tissue) [33–35], CCR7, CD62L (home to lymph nodes) [36, 37], CD278 (home to lung) [38], CD29 (home to the central nervous system) [39], CXCR4, CD122, CD132, and CD137 (home to bone marrow) [40–42], CXCR3 and CCR2 (home to inflamed tissues) [43, 44].

While low expression levels of ICOS and CD29 indicated that none of these B cell subsets mobilize to lung and central nervous system, it may be possible that these 2 markers as well as CD103 are not suitable for being used as an organ target marker for B cells. In contrast, some homing markers changed significantly during the course of dengue viral infection. Decreased levels of β7, CCR9, CCR7, CD62L, CXCR3 and CCR2 were observed in naïve and resting memory B cells. It indicated that the migrations of these B cell subsets to gut tissue, lymph nodes and inflamed tissues were reduced during dengue infection. The expression of CD62L on activated memory B cells was also obviously decreased whereas the expressions of CXCR4 and CCR2 were increased. It indicated that the migration of activated memory B cells to lymph node was reduced while their mobilization to bone marrow and inflamed tissues was elevated during dengue infection. Therefore, it might be possible that some activated memory B cells are denied re-entry into the lymph node and migrate into peripheral tissues sites of infection as well as bone marrow while they differentiated into plasmablasts/plasma cells.

In this study, an increased level of CCR7 was observed in tissue memory B cells. It indicated that the migration of these cells to lymph node was raised during dengue infection and their presence may be important in controlling immune response in lymph node. Although the function of this B cell subset is not well defined, tissue memory B cells (also called atypical memory B cells) present high-level expression of multiple inhibitory receptors on the cell surface. This expression results in B cell exhaustion. Therefore, this B cell subset proliferates and differentiates poorly in response to Ag-stimulated B cell [45, 46].

Since a marked increase of ASCs was observed during acute infection of dengue virus, the information on ASCs mobilization is important to understand B cell response during the course of disease. The results demonstrated that plasmablasts and plasma cells showed similar expression pattern for most homing molecules. While predominant frequencies of CD62L expression were observed on plasmablasts and plasma cells, remarkable higher frequencies of CCR7expression were also observed on both plasmablasts and plasma cells in dengue patients when compared with healthy individuals. However, the CCR7 expression levels in plasmablasts and plasma cells still lower than those observed in naïve and resting memory B cells. In contrast, high expression levels of CXCR4 were observed in both populations although no significant difference was observed when compared to healthy individuals. It indicated that down-regulation of CCR7 and up-regulation of CXCR4 may promote these subsets to leave from LNs and migrate to bone marrow. This observation was supported by a study showed that down-regulation of CCR7 on plasma cells can reduced the responsiveness to the B and T cell zone chemokine in lymph node whereas an increase chemotactic sensitivity to the CXCR4 ligand can promote the mobilization to bone marrow [47].

Previous study reported that CCR9 is mainly function in IgA-ASCs migrating to the small intestine during infection with rotavirus [48]. Interestingly, the expression levels of CCR9, on plasmablasts and plasma cells were increased during dengue infection. It indicated the potential migration of plasmablasts and plasma cells to gut tissues. Some evidence showed that dengue infection induced intestinal mucosal injury as demonstrated by increased serum levels of intestinal fatty acid binding protein which were used as a specific marker for mucosal injury [49]. Moreover, dengue infection in a mouse model revealed that inoculation of immune complexes formed with serotype cross-reactive antibodies resulted in increased vascular permeability in the small intestine [50]. Taken together, the potential migration of CCR9 expressing plasmablasts to the gut tissues may promote the formation of immune complexes which leads to severe small intestinal pathology. More interestingly, plasmablasts and plasma cells had significantly higher frequencies of CXCR3 and CCR2 expression in dengue patients than healthy individuals. They also showed highest frequencies when compared to other B cell subsets. It might be possible that high expressions of CXCR3 and CCR2 in both populations guide these effector cells to the inflamed areas. A previous study showed that CXCR3 and its ligands play a crucial role in the elimination and inflammatory responses to invading virus by leading plasmablasts or plasma cells to inflamed tissues [51]. CCR2 is also essential during monocytes transmigrate to inflamed tissues [44]. Interaction between CCR2 and its CC-chemokine ligand recruits monocytes to sites of infection and these monocytes can participate in the initial inflammatory response by producing tumor necrosis factor (TNF) and chemokines [44].

Therefore, it might be possible that elevation of these homing markers during infection promote plasmablasts and plasma cells or ASCs into those specific sites which might involve in protection or pathogenesis of dengue infection. Taken together, these data are very useful for our understanding of B cell subset distributions and organ mobilization based on the expression of homing molecules on different B cell subsets during the course of dengue viral infection.

## Conclusions

This study highlights the alteration of B cell subsets including naïve, resting, activated, and tissue memory cells as well as antibody secreting cells (ASCs) in pediatric dengue-infected patients during acute infection. The study found that the frequency of naïve B cells was markedly low, whereas that of ASCs was significantly increased. Moreover, results suggest that the change was not related to disease severity. It is also interesting to discover that the massively increased numbers of ASCs during acute infection expressed tissue homing markers, such as CXCR3 and CCR2, suggesting that these ASCs may distribute to inflamed tissues and various organs. Our findings, therefore, are very useful for better understanding of B cell subset distribution and organ mobilization according to homing molecule expression on different B cell subsets during the course of dengue viral infection.
